# Lack of Vertical Transmission of Grapevine Red Blotch Virus by *Spissistilus festinus* and Sex-Associated Differences in Horizontal Transmission

**DOI:** 10.3390/insects15121014

**Published:** 2024-12-21

**Authors:** Victoria J. Hoyle, Mackenzi Schultz, Elliot J. McGinnity Schneider, Brandon G. Roy, Marc Fuchs

**Affiliations:** 1 School of Integrative Plant Science, Plant Pathology and Plant-Microbe Biology, Cornell University, Geneva, NY 14456, USA; ejm372@cornell.edu (E.J.M.S.); bgr36@cornell.edu (B.G.R.); mf13@cornell.edu (M.F.); 2 College of Agriculture, Food and Natural Resources, University of Missouri, Columbia, MO 65203, USA

**Keywords:** grapevine red blotch virus, *Spissistilus festinus*, transovarial transmission, dispersal behavior, sex-associated transmission

## Abstract

Grapevine red blotch is an emerging disease that threatens vineyard productions in North America. Grapevine red blotch virus (GRBV, species *Grablovirus vitis*, genus *Grablovirus*, family *Geminiviridae*), the causal agent of red blotch disease, is transmitted by *Spissistilus festinus* (Hemiptera: Membracidae) in a circulative, non-propagative mode. To gain new insight into GRBV-*S. festinus* interactions, we delved into vertical transmission and documented a lack of transovarial transmission. In addition, we investigated *S. festinus* sex differences in the horizontal transmission of GRBV by creating small arenas with 30 detached trifoliates of common snap bean, an experimental host of GRBV, and a preferred feeding host of *S. festinus*. Tracking the movement of viruliferous males, females, or a combination of the two sexes over two weeks in replicated experiments demonstrated that male *S. festinus* dispersed more than females with specimens of both sexes predominantly grouping together on trifoliates spatially surrounding the trifoliate onto which they were released. These behaviors resulted in a greater rate of GRBV transmission by *S. festinus* males (17%, 20 of 120) than females (4%, 5 of 120) or mixed-sex cohorts (9%, 17 of 180). In arenas with aviruliferous *S. festinus* and one (single) or four (hotspot) GRBV-infected trifoliates out of 30 total trifoliates, a higher GRBV transmission rate by males was confirmed in both single infection (50%, 30 of 60) and hotspot infection (83%, 50 of 60) arenas than by females in single infection (35%, 21 of 60) and hotspot infection (67%, 40 of 60) arenas. These findings highlighted sex-associated differences in the transmission of GRBV by *S. festinus* and a positive correlation between the initial virus prevalence and the rate of transmission. Finally, the secondary spread of GRBV resulted primarily from *S. festinus* dispersal by walking or jumping. Together, these unique GRBV transmission features support the need to characterize dispersal behaviors of *S. festinus* in vineyard ecosystems.

## Simple Summary

Grapevine red blotch virus (GRBV, species *Grablovirus vitis*, genus *Grablovirus*, family *Geminiviridae*) is the first virus of grapevine to be transmitted by a treehopper vector, *Spissistilus festinus*, the three-cornered alfalfa hopper. Hypotheses for GRBV transmission have been by analogy with other viruses in the family *Geminiviridae.* In this study, we investigated novel attributes of GRBV transmission by *S. festinus* and determined the potential for vertical transmission and the differences in horizontal transmission by male and female *S. festinus* in a greenhouse. We found a lack of transovarial transmission, meaning females did not pass GRBV along to their offspring, and we found that males are overall greater contributors to GRBV spread than females. Moreover, we documented that *S. festinus* primarily spread GRBV in a walking and jumping dispersal pattern.. These results suggest a need for future studies in vineyard ecosystems to understand the sex-associated transmission of GRBV.

## 1. Introduction

Vineyards in North America have been devastated by the emergence of grapevine red blotch disease since the early 2010s [1]. Grapevine red blotch virus (GRBV, species *Grablovirus vitis*, genus *Grablovirus*, family *Geminiviridae*) is the causal agent of the disease [2]. Symptoms include delays in fruit ripening, reduction in fruit quality, and alteration of wine composition and sensory attributes [3,4]. The resulting impacts can vary annually but can affect vineyard profitability with up to USD 68,548 losses per hectare over the 25-year lifespan of a vineyard [5]. Isolates of GRBV belong to two phylogenetic clades for which the genetic diversity can differ by up to 9.1% [6].

GRBV is transmitted by *Spissistilus festinus* [Say, 1830] (Hemiptera: Membracidae)*,* the three-cornered alfalfa hopper [7,8]. GRBV isolates of phylogenetic clades 1 and 2 are transmissible by *S. festinus* [7]. Transmission of GRBV by this treehopper species is circulative and non-propagative, and thus, GRBV must transit through the body of *S. festinus* and reach the salivary glands before it can be transmitted upon feeding through the salivary canals within the stylets [7]. It is unknown whether GRBV can transverse other barriers beyond the gut and salivary glands within the bodies of *S. festinus*, such as the reproductive organs.

*Spissistilus festinus* is a dimorphic insect, with distinct male and female forms and five instar development stages [9]. Within vineyard ecosystems, more males than females have been observed among *S. festinus* populations [9,10], but it is not known how difference in sex-specific abundances may impact the spread of GRBV. In addition, males have been demonstrated to have a greater flight capacity in comparison with females under laboratory conditions [11]. It is not known if such a differential dispersal behavior of *S. festinus* males and females affects the spread of GRBV.

The aim of our study was to assess the vertical transmission of GRBV by *S. festinus* and characterize sex-associated differences in horizontal transmission using dispersal and behavioral assays combined with transmission assays with either aviruliferous or viruliferous *S. festinus* specimens. By analogy with other geminiviruses [12,13,14], we hypothesized a lack of transovarial GRBV transmission. We also hypothesized that *S. festinus* male dispersal results in a greater rate of GRBV transmission, particularly from a disease focus. Here, we present the results of replicated transmission assays and discuss our findings with regard to red blotch disease management strategies.

## 2. Materials and Methods

### 2.1. Plant Materials

Healthy snap beans *(Phaseolus vulgaris* cv. ‘Hystyle’) and alfalfa (*Medicago sativa*) were used as a rearing host of *S. festinus*. Alfalfa plants were also used as gut-clearing material and snap bean plants were used as recipient plant material in the transmission assays. Snap bean and alfalfa plants were set and maintained in greenhouses and later moved to controlled environmental chambers with the following growing conditions: 25 °C, 16 h:8 h light:dark photoperiod, and 80% relative humidity.

### 2.2. Snap Bean Inoculations with GRBV

Snap bean trifoliates were used as GRBV donor material in transmission assays. Trifoliates were derived from two to three-week old snap bean plants that were pinprick-inoculated in 2-cm intervals along the stems and petioles and three to four inches from the soil line. Inoculations were performed using sterile dissecting needles swabbed with recombinant *Agrobacterium tumefaciens* harboring an infectious clone of GRBV isolate NY175 (phylogenetic clade 1) or isolate NY358 (phylogenetic clade 2), as previously described [7,15].

### 2.3. Spissistilus festinus

*S. festinus* specimens were derived from a colony population collected in Yolo, San Joaquin and Fresno counties in California. They were maintained in a controlled environmental chamber at Cornell AgriTech in Geneva, NY with rearing conditions set at 26 °C, 14:10 light/dark photoperiod, and 70–80% relative humidity [16]. Individual insects were sexed upon molt into adulthood and separated into males and females before use in experimentation.

### 2.4. Transovarial Transmission Assays

**Figure 1:**
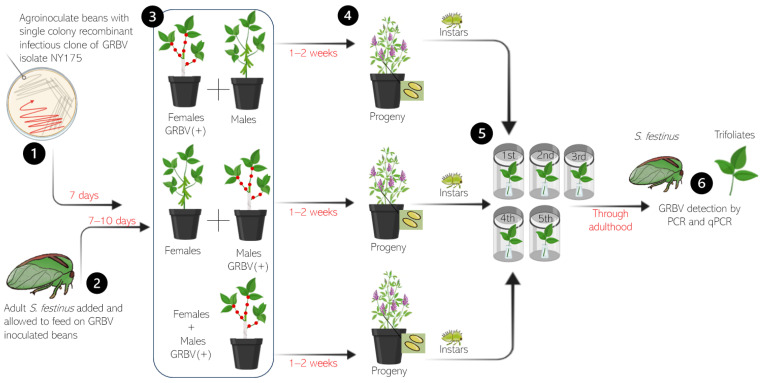
Schematic representation of the transovarial transmission assays with (1) grapevine red blotch virus (GRBV) inoculation of snap bean plants, (2) *Spissistilus festinus* from the colony being exposed to GRBV inoculated snap bean plants as associated with three treatments, (3) three treatments and 1–2 week acquisition access period, (4) the mixing and mating of males and females on alfalfa through the emergence of progeny, (5) the release of 1st instar through adult progeny on healthy, detached snap bean trifoliates, (6) testing of plants and insects for GRBV using multiplex PCR and qPCR. Recipient tissues were collected from detached chambers for GRBV testing and replaced at every molt. The artwork was produced using the program BioRender (Toronto, ON, Canada).

Cohorts of either newly emerged adult *S. festinus* males, females, or a combination of the two were exposed to GRBV-inoculated bean plants pinpricked with GRBV isolate NY175 (phylogenetic clade 2) for 1–2 weeks. Snap bean stems not inoculated with GRBV were wrapped with cheese cloth to encourage feeding by *S. festinus* on GRBV-inoculated areas [15]. Next, cohorts were moved to alfalfa and joined by cohorts of the opposite sex, not previously exposed to GRBV-inoculated plants for two weeks to reproduce and lay eggs. After two weeks, adults were removed, and alfalfa plants were monitored for the emergence of progeny. First instars through newly emerged adult *S. festinus* were moved in cohorts of 5–7 onto uninoculated bean trifoliates enclosed in a clear polypropylene 950-mL container (cat. no. PK32T, Fabri-Kal, Kalamazoo, MI, USA) sealed with a polypropylene donut lid carrying a nylon screen (BugDorm 5002, MegaView Science, Taichung, Taiwan) to monitor possible GRBV transmission. After each molt, nymphs were moved to new healthy detached trifoliates. All *S. festinus* were collected after their final molt and tested for GRBV by multiplex PCR and qPCR. Similarly, bean trifoliates were collected for each treatment and tested for GRBV by multiplex PCR and qPCR (Figure 1).

### 2.5. Dispersal Assays of Viruliferous S. festinus and Impact on GRBV Transmission

**Figure 2:**
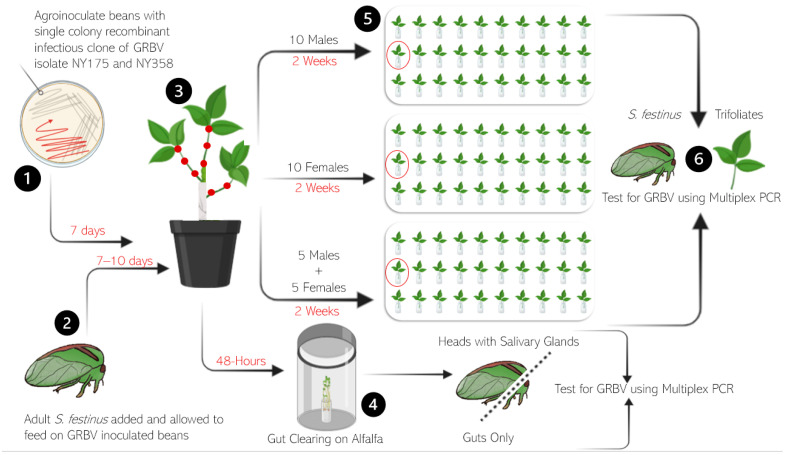
Schematic representation of the *Spissistilus festinus* dispersal experiments on detached snap bean trifoliates with (1) grapevine rep blotch virus (GRBV) inoculation of snap bean plants, (2) *S. festinus* from the colony being exposed to GRBV inoculated beans, (3) the acquisition access period, (4) the gut clearing of *S. festinus* on alfalfa plantlets to test for acquisition, (5) behavior and movement tracking in separate arenas, and (6) testing of plants and insects for GRBV using multiplex PCR. The artwork was produced using the program BioRender (Toronto, ON, Canada).

Cohorts of either newly emerged adult *S. festinus* males, females, or a combination of the two were exposed to GRBV-inoculated bean plants pinpricked with GRBV isolate NY358 (phylogenetic clade 1) or NY175 (phylogenetic clade 2). Snap bean stems not inoculated with GRBV were wrapped with cheese cloth to encourage feeding by *S. festinus* on GRBV-inoculated areas. *S. festinus* fed on inoculated snap bean tissue in cages (BugDorm 6E610 Insect Rearing Cage, MegaView Science, Taichung, Taiwan) separated by treatment type for 7–10 days. Arenas were set up containing 30 healthy excised bean trifoliates in parafilmed water vials spaced out in 2–3-inch intervals, with 10 trifoliates per row (Figure 2). Ten GRBV-exposed *S. festinus* pertaining to each treatment type (all males, all females, or 50/50 mix) were moved on alfalfa enclosed in a clear polypropylene 950-mL container (cat. no. PK32T, Fabri-Kal, Kalamazoo, MI, USA) sealed with a polypropylene donut lid carrying a nylon screen for 48-h for a gut-clearing period before being released on the first trifoliate of the second row in their arenas (Figure 2). To test for GRBV acquisition, subsets of 3–5 *S. festinus* per treatment were collected following the 48-h on alfalfa and dissected for heads with salivary glands and guts to be tested for GRBV by multiplex PCR. The experiments were replicated 2–3 times.

Following a 48-h gut clearing on alfalfa, *S. festinus* from their respective treatments were released into arenas (BugDorm-4S4590DH Specimen Handling Cage, MegaView Science, Taichung, Taiwan) containing 30 healthy bean trifoliates in water, spaced 2–3 inches apart. The dispersal behavior of *S. festinus* was recorded twice daily, in the morning and afternoon, for two weeks, noting the presence and absence of *S. festinus* on each trifoliate and on the cage, accounting for all ten *S. festinus* in each cage at each time point. Following a two-week period of behavioral movement tracking, *S. festinus* and plant tissues were collected and tested for GRBV using multiplex PCR and qPCR (Figure 2). Plant tissues were processed into the petiole, leaf-petiole junction, and leaf midrib prior to nucleic acid extraction to account for differential hopper feeding behavior. The experiments were replicated 3–4 times. A trifoliate was considered positive if one or more tissue types, i.e., petiole, leaf-petiole junction, and/or midrib, tested positive for GRBV by multiplex PCR.

### 2.6. Feeding and Dispersal Behaviors of Spissistilus festinus

The feeding and movement behaviors of *S. festinus* were observed on snap bean trifoliates. Feeding behaviors were classified into two categories: probing (gentle feeding of trifoliate tissue with minimal signs of injury to the plant) and girdling (destructive, circular feeding of the trifoliate tissue, often cutting off its vasculature). Dispersal behaviors were classified into three categories: walking (movement along or across bean trifoliates, up and down, or side to side with legs remaining grounded), jumping (bounding motion to move multiple trifoliates away without flight), and flying (movement requiring the extension and flapping of wings, general upward until coming into contact with the cage).

### 2.7. Dispersal Assays of Aviruliferous S. festinus and Impact on Secondary Spread of GRBV

**Figure 3:**
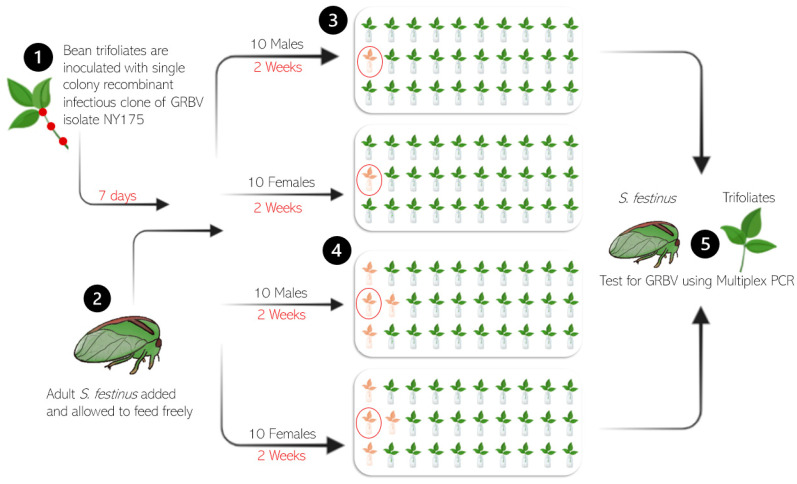
Schematic representation of transmission assays with grapevine red blotch virus (GRBV)-infected snap bean trifoliates and aviruliferous male and female *Spissistilus festinus* with (1) GRBV inoculation of snap bean trifoliates, (2) *S. festinus* from the colony released in each arena, (3) arenas where *S. festinus* was placed on a single trifoliate (red circle) with one red trifoliate (single infection) or (4) where *S. festinus* was placed on a single (red circle) of four red trifoliate (hot spot infection), and (5) testing of plants and insects for GRBV using multiplex PCR. The artwork was produced using the program BioRender (Toronto, ON, Canada).

Snap bean plants were agroinoculated using the aforementioned approach [7,15]. After at least one week, trifoliates were detached from snap bean plants agroinoculated with the GRBV isolate NY175 (phylogenetic clade 2) and were placed into water vials in an arena (BugDorm-4S4590DH Specimen Handling Cage, MegaView Science, Taichung, Taiwan) containing either a single GRBV-inoculated leaf at the first location of the second row (single infection point) or at three first positions plus the second position of the second row (hotspot) (Figure 3). All remaining excised leaves in each arena were healthy, uninoculated snap bean trifoliates. Ten colony-reared, non-viruliferous male or female *S. festinus* were released on the first trifoliate of the second row in their arenas (Figure 3) and were allowed to move around freely feeding for two weeks. Following a two-week period, *S. festinus* and plant tissues were collected and tested for GRBV using multiplex PCR (Figure 3). Snap bean tissue from the petiole, leaf-petiole junction, and midrib were processed separately. The experiments were replicated 3–4 times.

### 2.8. Nucleic Acid Isolation, Diagnostic GRBV Multiplex PCR, and Quantification of GRBV by qPCR

Genomic DNA was isolated from either plant tissue using the MagMAX™-96 A1/ND Isolation Kit (ThermoFisher Scientific, Waltham, MA, USA) or from *S. festinus* using the MagMAX™ DNA Multi-Sample Ultra Kit (ThermoFisher Scientific, Waltham, MA, USA) on a KingFisher™ instrument. To test for GRBV in plant tissue and *S. festinus* by PCR, primer pairs designed in the open reading frame (ORF) coding for the predicted coat protein (CP) gene and replication-associated (RepA) proteins were utilized to amplify the isolated genomic DNA [7]. DNA amplicons were visualized by agarose gel electrophoresis and UV illumination following GelRed^®^ staining (Biotium, Fremont, CA, USA).

For the quantification of GRBV in plant samples and *S. festinus* specimens by qPCR, each reaction was conducted in triplicate (three technical replicates) on a Biorad C1000 Touch thermocycler and utilized SYBR^®^ Green reagents (iTaq Bio-Rad Universal SYBR^®^ Green Supermix, Hercules, CA, USA). Testing plant tissue for GRBV by qPCR required primers pREP3v and pREP4v [17] and a primer pair designed in the bean housekeeping gene T197 coding a guanine nucleotide-binding protein beta subunit-like protein [8,18]. Negative controls included sterile water and nucleic acids isolated from healthy, excised bean trifoliates. Testing *S. festinus* for GRBV by qPCR required primers pREP3v and pREP4v [17] and *S. festinus* primers Sf18SFor and Sf18SRev [19]. Negative controls included sterile water and nucleic acids isolated from *S. festinus* from the colony maintained on the healthy snap bean.

### 2.9. Statistical Analysis

Statistical analyses were performed on the GRBV titers in plant tissue and insect specimens, according to qPCR Ct values with ANOVA in R Studio (version 4.4.1). Fold differences in GRBV titer were based on ΔΔCt values. Statistically significant differences between ΔΔCt expression fold change values (2(−ΔΔCt)) in excised leaves and adult *S. festinus* were determined using Tukey’s Honestly Significant Difference (HSD) test. To assess dispersal behavior and the pattern of influence for transmission, we utilized ANOVA and regression analysis. The significance level was set at α = 0.05 for all statistical analyses performed in this study.

## 3. Results

### 3.1. Transovarial Transmission of GRBV by Spissistilus festinus

The possibility of vertical transmission of GRBV by adult female *S. festinus* to their progeny was assessed by rearing a generation of *S. festinus* nymphs derived from parents exposed to GRBV-infected snap bean plants, confirmed for GRBV acquisition by multiplex PCR, and mated prior to being released on alfalfa, a non-host of GRBV [7], where oviposition occurred (Figure 1). First through fifth instar *S. festinus* were moved to healthy detached trifoliates to develop to adulthood, with trifoliates collected and replaced between each molt. No adult progeny was derived from any of the three treatment types (males, females, and 50/50 mix) (0%, 0/195) that tested positive for GRBV in PCR or qPCR (Table 1).

As expected, none of the snap bean trifoliates (0%, 0/324), to which the different instars or treehopper adults were exposed tested positive for GRBV in PCR or qPCR (Table 2). Together, these results revealed a lack of transovarial GRBV transmission by *S. festinus*.

### 3.2. The Dispersal Behavior of Male and Female Spissistilus festinus

**Figure 4:**
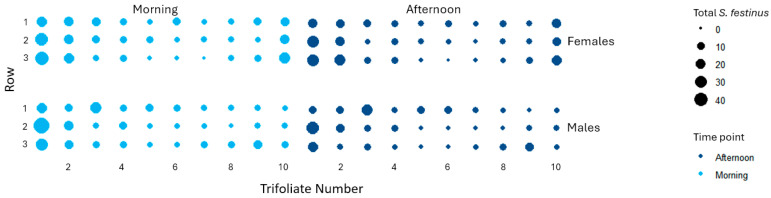
The cumulative distribution of *Spissistilus festinus* across 30 snap bean trifoliates in arenas with 10 males or 10 females, in the morning or afternoon, over two weeks, from four experimental replicates. The counts of *S. festinus* per trifoliate were derived from the total counts across all four replicates per time point.

**Figure 5:**
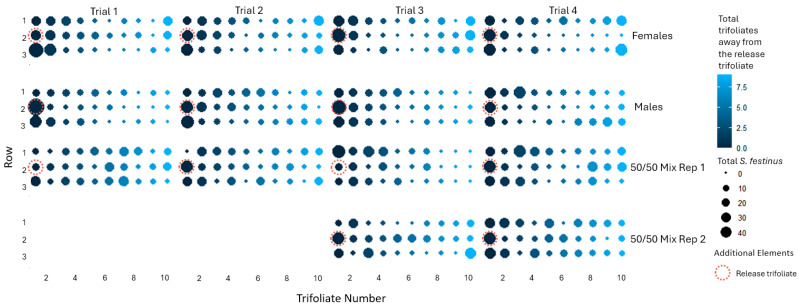
The cumulative distribution of *Spissistilus festinus* across 30 snap bean trifoliates in arenas with 10 males, 10 females, or mixed males (*n* = 5) and females (*n* = 5) over two weeks, from four experimental replicates (1–4). The darker the circle, the closer the column distance to the release point, and the larger the circle, the greater the total number of *S. festinus* present on that trifoliate over time. The counts of *S. festinus* per trifoliate were derived from a cumulative average of the morning and afternoon counts.

The dispersal behaviors of *S. festinus* were observed in arenas with either 10 males, 10 females, or a mix of both sexes (n = 5 + 5) in the mornings and afternoons, for two weeks, in four replicated experiments. Male *S. festinus* dispersed more than females, spending more time in the afternoons off trifoliates and on the cages, as illustrated by the overall small size dots for males compared with females (Figure 4). However, even though there was a reduction effect on total male *S. festinus* on trifoliates, this result was not statistically significant (*p* = 0.178). Specimens of both sexes predominantly grouped together over time on trifoliates surrounding the trifoliate onto which they were released in the arenas, suggesting prevalent spatiotemporal clustering (Figure 5).

**Figure 6:**
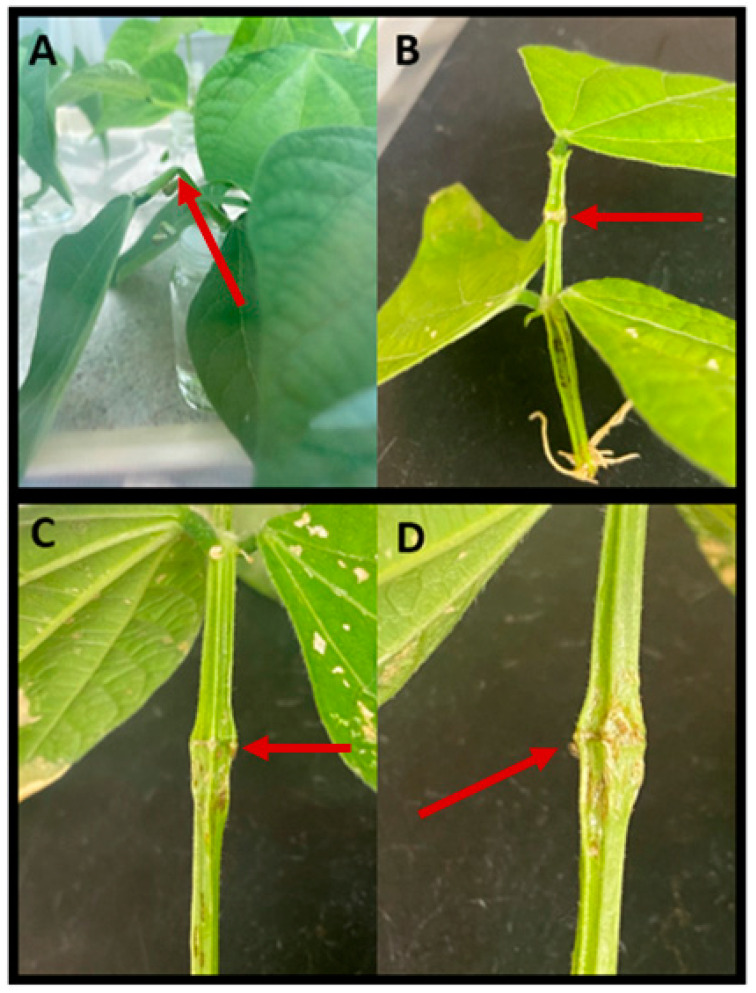
The different locations of girdling behaviors observed by male and female *Spissistilus festinus* in arenas with both sexes present. Male girdling behaviors were often observed around the time of mating (**A**) and would result in vascular cut-off along the petiole leaf junction (**B**). Female girdling behaviors were often observed around the time of oviposition and were right above the oviposition sites (**C**,**D**). This behavior is characteristic of mothering and could be enacted to encourage proper feeding of nymphs upon emergence (**D**). Red arrows indicate girdled snap bean tissue.

Observations of the behaviors of *S. festinus* over time indicated three distinct types of movement within arenas: walking, jumping, and flying. Similarly, two distinct feeding behaviors of *S. festinus* were noticed: probing and girdling. However, distinct feeding behaviors were only observed in arenas with mixed males and females with *S. festinus* males girding the petiole leaf junction (Figure 6A,B) and *S. festinus* females girdling the petiole above the oviposition site (Figure 6C,D).

### 3.3. Dispersal of Viruliferous Male and Female Spissistilus festinus and Their Impact on Grapevine Red Blotch Virus Transmission

Dispersal assays were performed in bean trifoliate arenas with viruliferous *S. festinus* males and females that were exposed to snap bean plants infected with a GRBV isolate of phylogenetic clade 1 or 2. After two weeks of release in the arenas, all *S. festinus* specimens and the 30 trifoliates were removed from each arena and tested for GRBV. In inoculated plants that served as virus donors, a slightly lower GRBV titer was observed in clade 1- (Ct values of 23–27) than in clade 2-inoculated (Ct values of 20–23) plants. However, regardless of the GRBV isolate, a greater rate of GRBV transmission was observed in male-only cages (17%, 20/120) in comparison with female-only cages (4%, 5/120) or mixed cages (9%, 17/180) (*p* = 0.005) (Table 3).

**Figure 7:**
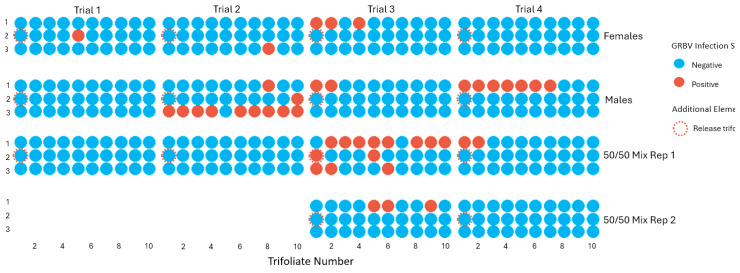
Transmission of grapevine red blotch virus (GRBV) from bean trifoliate to bean trifoliate by viruliferous male (*n* = 10), female (*n* = 10), or a mix of male (*n* = 5) and female (*n* = 5) *Spissistilus festinus*, over two weeks, across four replicated arenas. *S. festinus* were deposited on a single trifoliate (dotted circle) located at one edge of each arena. Data show GRBV-infected trifoliates (red dot) and noninfected trifoliates (blue dots), as determined by PCR.

Analyzing the distribution of GRBV-infected bean trifoliates in each arena indicated that most of them were near the single release trifoliate, suggesting a clustering behavior of the viruliferous *S. festinus*, though sometimes infected trifoliates were away from the single-release trifoliate (Figure 7). In arenas with viruliferous *S. festinus* males only, a within-row distribution pattern was apparent (Figure 7).

To determine if the clustering behavior of viruliferous *S. festinus* males and females influence horizontal transmission of GRBV, a logistic regression model was conducted to determine if the count of total *S. festinus* present on a trifoliate influenced the likelihood of a trifoliate to test positive for GRBV, across the different treatments. Though the log-odds of infection increased with additional *S. festinus* presence on trifoliates, there was no significant increase in GRBV infection across treatments (*p* = 0.160). However, the inclusion of females did reduce the likelihood of transmission (*p* = 0.00143). These results demonstrated a difference in transmission among the two sexes of *S. festinus* and warrant further analysis to draw a conclusion on whether this influence is behavioral or biological.

### 3.4. Dispersal of Aviruliferous Spissistilus festinus Males and Females and Impact on Secondary Grapevine Red Blotch Virus Spread in Arenas with Single and Multiple Initial Infections

In experiments with aviruliferous *S. festinus* and a single or four (hotspot) GRBV-infected snap bean trifoliates at one edge of each arena (Figure 3), GRBV transmission occurred at a higher rate with males in both single (50%, 30/60) and hotspot (83%, 50/60) arenas than with females in single (35%, 21/60) and hotspot (67%, 40/60) arenas (Table 4).

**Figure 8:**
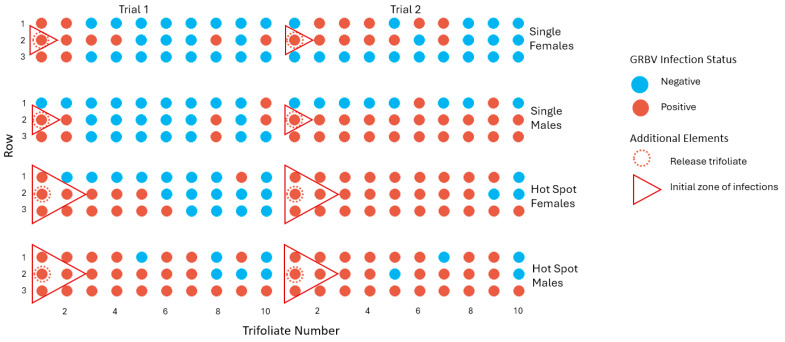
Transmission of grapevine red blotch virus (GRBV) from snap bean trifoliate to snap bean trifoliate by aviruliferous male (second and fourth row arenas) or female (first and third row arenas) *Spissistilus festinus*, over two weeks, in arenas with single (first and second row arenas) or four (hotspot) (third and fourth row arenas) GRBV-infected snap bean trifoliates (red triangle). Insects were deposited on a single trifoliate (dotted circle) located at one edge of each arena. Data show GRBV-infected trifoliates (red dot) and noninfected trifoliates (blue dots), as determined by PCR.

Analyzing the distribution of newly infected GRBV-infected trifoliates in single infection arenas revealed a clustering near the virus donor trifoliate upon which *S. festinus* specimens were released and a within-row predominant spread (Figure 8). These results may result from a walking and jumping movement of *S. festinus* that acquired GRBV by feeding on the single infected trifoliate. In hotspot arenas, the distribution of newly infected GRBV-infected trifoliates confirmed a predominant walking and jumping movement behavior of *S. festinus* (Figure 8). These results revealed the secondary spread of GRBV primarily in relation to walking and jumping movement patterns of *S. festinus*.

**Figure 9:**
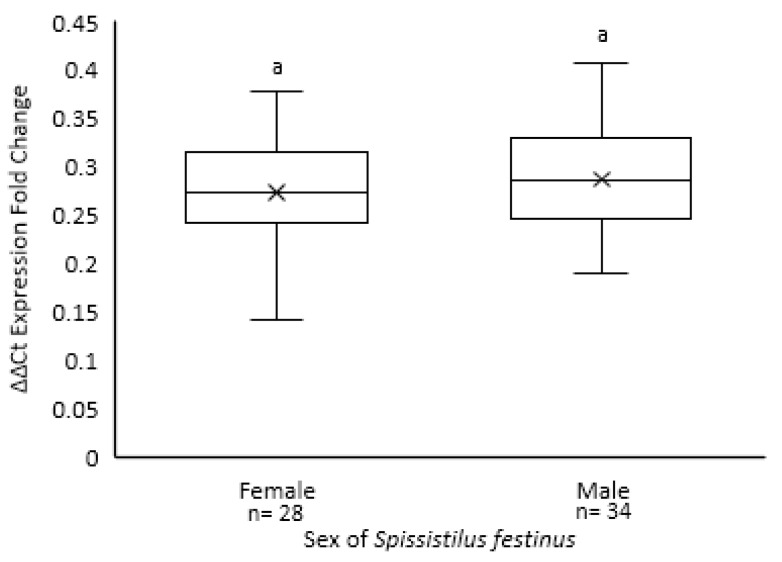
Quantification of grapevine red blotch virus in female and male *Spissistilus festinus* used in single and hot spot infection assays by qPCR. Data show the ΔΔCt expression fold change values (2^(−ΔΔCt)^). The (x) represents the mean expression fold change. Error bars indicate standard error of the mean. Lowercase lettering indicating insignificant differences between 2^(−ΔΔCt)^ values, as determined by Tukey’s honestly significant difference test (*p* < 0.05), are shown above standard error bars.

Testing of *S. festinus* at the completion of the dispersal experiments revealed a similar acquisition rate of GRBV by males (88%, 35/40) and females (78%, 28/36), with no statistically significant differences in the GRBV titer in a randomly selected subset of these specimens, as shown by qPCR (*p* = 0.360) (Figure 9). These findings highlighted sex-associated behavioral differences in the transmission of GRBV by *S. festinus* and a positive correlation between the initial virus prevalence and the rate of transmission.

## 4. Discussion

In this study, we documented a lack of vertical transmission of GRBV by *S. festinus* using transmission assays with snap bean, a rearing host of *S. festinus*, and a pseudo systemic host of GRBV [7]. These results are consistent with similar reports on viruses in the family *Geminiviridae* [12,13,14]. Therefore, the absence of transovarial transmission of GRBV by the progeny of viruliferous *S. festinus* validates our hypothesis.

We further investigated the dispersal of *S. festinus* males and females in small arenas with snap bean trifoliates. Following the release of cohorts of 10 specimens on a single trifoliate, both sexes were shown to preferentially aggregate near the trifoliate of release, though males were more likely to disperse in the afternoons, returning over time near to their trifoliate of release. This differential behavioral dispersal of *S. festinus* resulted in a greater rate of transmission of GRBV by males in comparison to females, and a clustering of newly infected trifoliates near the single trifoliate upon which insects were released. Similarly, males contributed to more efficient secondary spread of GRBV than females, though both sexes had similar rates of virus acquisition. In addition, a positive correlation was found between initial virus prevalence and transmission rate. Movement behaviors of *S. festinus* based on walking and jumping explained the patterns of secondary spread. It will be interesting to monitor the behavior and dispersal of *S. festinus* males and females in vineyard ecosystems. Such studies should help to facilitate a better understanding of the unique epidemiological attributes of red blotch disease previously reported [3,20,21,22,23].

Male *S. festinus* have a greater flight capacity in comparison with females (on average 570.22 m vs. 239.57 m) [11]. It is not known if this sex-based difference may affect secondary spread of GRBV in vineyard ecosystems. However, based on our findings, it is reasonable to anticipate that *S. festinus* males are major contributors to the spread of GRBV in vineyards. Furthermore, more males than females are caught on sticky cards in vineyards [10] and alfalfa fields [24], although a balanced sex-ratio was observed with the use of sweep netting in alfalfa fields [24]. This suggests differences in male and female dispersal behaviors. In this study, we observed a large proportion of afternoon flights by males, coupled with a greater rate of GRBV transmission, suggesting that sex-associated differences in transmission are influenced by behavior.

An overabundance of *S. festinus* and feeding was previously observed to negatively impact the transmission success of GRBV, likely due to the girdling of grapevine tissue [22]. It is possible that feeding behaviors and larger aggregations of *S. festinus* females could result in lower rates of transmission due to extensive feeding damages. In contrast, *S. festinus* male dispersal and potential probing rather than girdling behaviors during this time could promote the spread of GRBV. This should be monitored further to better understand *S. festinus* feeding and/or reproduction behaviors by, for instance, using electrical penetration graph technologies.

In this study, the transmission of GRBV by *S. festinus* was low (10%, 42/420) (Table 3) with viruliferous specimens when combining data obtained with males, females, and a 50/50 mix across replicated experiments. Nonetheless, this transmission rate is consistent with that previously observed in transmission assays in the greenhouse (4–42%) on wine grape cultivars [7,8] and vineyards (3–28%) [3,20,21,22,23]. In contrast, a high rate of transmission of GRBV (56%, 123/220) (Table 4) was observed by both *S. festinus* sexes when aviruliferous specimens had to acquire the virus first on infected trifoliates and transmit it to healthy trifoliates in the arenas. This high rate of transmission suggests that *S. festinus* were almost immediately feeding on infected trifoliates upon release; it should be considered that a 6-day acquisition access period is needed for bean-to-bean transmission [7] and our experiments lasted 14 days. This marked difference in GRBV transmission by viruliferous and aviruliferous *S. festinus* could be related to the age of the insects, as discussed below.

In experiments using viruliferous insects, very few *S. festinus* tested positive for GRBV (15%, 9/60), whereas in experiments using aviruliferous hoppers from arenas with single (70%, 28/40) or hotspot infections (97%, 35/36), *S. festinus* tested positive for GRBV at the completion of the transmission assays. These differences in virus acquisition could be due to the age of the *S. festinus* specimens when they were released into the arenas. Experiments with viruliferous *S. festinus* relied on specimens that were approximately two weeks older than those used in experiments with aviruliferous specimens. This is around the time when female *S. festinus* lay their first eggs if they had an opportunity to mate. It is likely that this important step within the life cycle of adult *S. festinus* alters movement and feeding behaviors, thus reducing the rate of virus acquisition and transmission. In addition, more *S. festinus* specimens, primarily males, died due to old age, during the experiments with viruliferous insects, impacting the rate of virus transmission.

Epidemiological studies have documented an edge effect in the spatiotemporal distribution of GRBV-infected vines in vineyards at an early stage of a red blotch disease epidemic [3,21]. Diseased vines clustering at the edge of a vineyard could serve as a transmission hotspot to facilitate secondary spread of GRBV. Based on our dispersal assays, there was an increase in GRBV-positive *S. festinus* as the size of the inoculum increased. Similarly, there was a positive association between the initial virus prevalence and the rate of GRBV spread. Such results are consistent with the edge effect with the vineyard observations.

In vineyard ecosystems, *S. festinus* interact with free-living vines, using them as a feeding and possible overwintering host [10]. If the behavior of *S. festinus* in vineyards is similar to the behavior observed in this study, *S. festinus* visiting vineyards, including diseased vineyards, could return to their preferred feeding and reproductive hosts in natural habitats, potentially resulting in a build-up of virus inoculum in riparian areas, as previously suggested [25]. More work is needed to confirm this potential.

The primary source of GRBV in vineyards is planting material. Newly established grapevines that are infected by GRBV form a source of inoculum for secondary spread, which can be extensive if virus incidence is high [20,22], although, in this study, *S. festinus* is confirmed as an inefficient vector of GRBV and transmission rates are not as high as previously described [7,8]. Based on the polyphagous nature of *S. festinus* and their preferred feeding and reproductive hosts in natural and riparian habitats rather than in vineyards, we cannot stress enough the importance of planting certified, clean planting stocks that have been extensively tested for the absence of GRBV, and the roguing of diseased vines or removal of entire vineyards if disease incidence is below or above 30%, respectively [5]. To refine these disease management responses, more work is needed to characterize the sex ratio of male and female *S. festinus* in vineyards, and their dispersal in relation to disease epidemiology.

## Figures and Tables

**Table 1:** Lack of transovarial transmission of grapevine red blotch virus (GRBV) by *Spissistilus festinus*. The number of *S. festinus* progenies from females and males that were exposed to GRBV-infected plants and tested negative for GRBV by multiple PCR or qPCR following transfer to healthy detached bean trifoliates at all developmental stages to live through adulthood (Ad) is indicated.

Treatment	1st	2nd	3rd	4th	5th	Ad.
Females	0/4	0/5	0/4	0/13	0/17	0/20
Males	0/1	0/5	0/9	0/14	0/19	0/13
50/50 Mix	0/3	0/5	0/13	0/14	0/17	0/19
Total GRBV Positive Progeny	0/8 (0%)	0/15 (0%)	0/26 (0%)	0/41 (0%)	0/53 (0%)	0/52 (0%)

**Table 2:** No detectable grapevine red blotch virus (GRBV) in snap bean trifoliates following feeding by the nymphal progeny of *Spissistilus festinus* females and males that were exposed to GRBV-infected plants across all developmental stages and through adulthood (Ad), as shown by multiplex PCR or qPCR. Fractions represent the number of trifoliates that tested positive for GRBV over the total number of trifoliates tested.

	Treatment																	
	Males						Females						50/50 Mix					
	1st	2nd	3rd	4th	5th	Ad.	1st	2nd	3rd	4th	5th	Ad.	1st	2nd	3rd	4th	5th	Ad.
1st	0/6						0/6						0/6					
2nd	0/6	0/6					0/6	0/6					0/6	0/6				
3rd	0/6	0/6	0/6				0/6	0/6	0/6				0/6	0/6	0/6			
4th	0/6	0/6	0/6	0/6			0/6	0/6	0/6	0/6			0/6	0/6	0/6	0/6		
5th	0/6	0/6	0/6	0/6	0/6		0/6	0/6	0/6	0/6	0/6		0/6	0/6	0/6	0/6	0/6	
Ad.	0/6	0/6	0/6	0/6	0/6	0/6	0/6	0/6	0/6	0/6	0/6	0/6	0/6	0/6	0/6	0/6	0/6	0/6

**Table 3:** Transmission of grapevine red blotch virus (GRBV) isolates NY358 (phylogenetic clade 2) and NY175 (phylogenetic clade 1) by male and female *Spissistilus festinus*. The total number of snap bean trifoliates that tested positive for GRBV by PCR or qPCR following a 10-day acquisition access period and a 2-week inoculation access period on bean trifoliates in arenas of males, females, and a mix of males and females is indicated. Lowercase letters represent statistical significance across treatments (*p* < 0.05).

	Males	Females	50/50 Mix
Clade 2	11/60	2/60	0/60
Clade 1	9/60	3/60	17/120
Total	20/120 (17%) ^a^	5/120 (4%) ^b^	17/180 (9%) ^ab^

**Table 4:** Rate of grapevine red blotch virus transmission by *Spissistilus festinus* males and females in arenas with single or hotspot infections. Uppercase letters represent statistical significance across treatments and lowercase letters represent statistical significance between sexes (*p* < 0.05).

	Males	Females	Total
Single Infection Arena	30/60 (50%) ^Aa^	21/60 (35%) ^Aa^	51/120 (43%) ^A^
Hotspot Infection Arena	50/60 (83%) ^Ba^	40/60 (67%) ^Bb^	90/120 (75%) ^B^
Total GRBV-Infected Trifoliates	80/120 (67%) ^a^	61/120 (51%) ^b^	

## Data Availability

The original contributions presented in this study are included in the article. Further inquiries can be directed to the corresponding author.
